# Gastric juice MicroRNAs as biomarkers for functional dyspepsia: an observational case-control study

**DOI:** 10.3389/fmed.2025.1636825

**Published:** 2025-08-26

**Authors:** Radu A. Farcas, Teodora Surdea-Blaga, Ştefan Popa, Flaviu Rusu, Alexandra Chira, Cristina Sabo, Vlad-Ionuţ Nechita, Liviuţa Budişan, Oana Zanoaga, Ioana Berindan-Neagoe, Ştefan Strilciuc, Dan L. Dumitrascu

**Affiliations:** ^1^2^nd^ Department of Internal Medicine, “Iuliu Haţieganu” University of Medicine and Pharmacy, Cluj-Napoca, Romania; ^2^Department of Medical Education – Medical Informatics and Biostatistics, “Iuliu Haţieganu” University of Medicine and Pharmacy, Cluj-Napoca, Romania; ^3^Genomics Department, MEDFUTURE Institute of Biomedical Research, Iuliu-Hatieganu University of Medicine and Pharmacy, Cluj-Napoca, Romania; ^4^Doctoral School, Iuliu Hatieganu University of Medicine and Pharmacy, Cluj-Napoca, Romania; ^5^Academy of Medical Sciences, Bucharest, Romania

**Keywords:** functional dyspepsia, epigastric pain syndrome, microRNAs, postprandial distress syndrome, biomarker, miR

## Abstract

**Background and aims:**

MicroRNAs (miRNAs, miRs) are stable RNA molecules that regulate gene expression and hold promise as biomarkers. Functional dyspepsia (FD), a complex gastrointestinal disorder, is characterized by motility dysfunction, visceral hypersensitivity, and gut microbiome alterations. Given the limitations of current diagnostic markers, miRNAs may offer novel insights for diagnosis and risk stratification.

**Methods:**

We conducted an observational case–control study involving 28 FD patients (14 with epigastric pain syndrome [EPS], 14 with postprandial distress syndrome [PDS]) and 22 healthy controls (HC). All subjects underwent gastroscopy with gastric juice collection. Quantitative real-time PCR was used to measure levels of selected miRNAs (miR-21-5p, miR-155-5p, miR-203a) in gastric fluid, with miR-16 and U6 as endogenous controls. Fold-change in miRNA expression was calculated. We compared miRNA levels between groups and between FD subtypes and assessed correlations with symptom severity (using the Functional Dyspepsia Symptom Diary scores). Statistical significance was determined by non-parametric tests, with *p* < 0.05 as threshold.

**Results:**

Gastric juice miR-21-5p was significantly elevated in FD patients compared to controls (FD: 19.98 ± 91.56 fold-change vs. HC: 2.63 ± 3.30 fold-change *p* = 0.00774). In contrast, miR-155-5p and miR-203a levels did not differ significantly overall between FD and healthy groups. However, within FD, PDS patients showed a distinct profile: markedly higher miR-21 and miR-155 but lower miR-203 relative to EPS patients. MiRNA levels correlated with symptom patterns: miR-21 and miR-155 levels were inversely correlated with epigastric pain intensity (τ = −0.517 and −0.317, respectively) but positively correlated with postprandial fullness and early satiety (τ up to 0.523, *p* < 0.01). Conversely, miR-203 showed direct correlation with epigastric pain (τ = 0.317, p = 0.023) and inverse correlation with fullness (τ = −0.523, *p* < 0.01). A history of Helicobacter pylori infection was associated with a significant reduction in gastric juice miR-203 levels (*p* ∼ 0.03).

**Conclusion:**

FD patients might have altered gastric juice miRNA profiles, notably an upregulation of miR-21, and distinctive differences between PDS and EPS subtypes. The PDS subtype is characterized by a high-miR-21 and miR-155 and low-miR-203 signature, whereas the opposite pattern is observed in EPS. These miRNA alterations align with the symptomatology of FD subtypes and may reflect underlying pathophysiological differences. Gastric juice miRNAs could serve as minimally invasive biomarkers for FD, aiding in differentiating functional subgroups and potentially guiding targeted therapies. Further studies are warranted to confirm this findings and establish their diagnostic utility and role in FD pathogenesis.

## 1 Introduction

MicroRNAs (miRNAs, miRs) are small, endogenous RNA molecules that modulate gene expression at the post-transcriptional level through interactions with target mRNAs, leading to either inhibition of translation or mRNA degradation (1–3). Their pronounced stability, efficient release into extracellular compartments, and reliable detection even at low concentrations make them potential biomarkers of various diseases, including functional dyspepsia (FD) (3).

Functional dyspepsia is a common gastrointestinal disorder that not only severely impacts patients’ quality of life but also imposes a significant economic burden on healthcare systems. The clinical management of FD is challenging due to its various presentation, characterized by dysregulated gastric motility, heightened visceral sensitivity, and emerging evidence of gut microbiome alterations (4–8). The need for biomarkers that can guide treatment decisions, track disease progression, and forecast clinical outcomes in disorders of gut-brain interactions is urgent. While current research has largely focused on protein-based markers and drinking tests for real-time clinical decision-making, microRNAs may enable clinicians to refine risk stratification and better characterize the underlying pathophysiological heterogeneity in FD (9, 10).

Several gastric juice microRNAs have been shown to be promising biomarker candidates in functional dyspepsia (11).

This study aims to evaluate the miRNA expression profiles in patients with FD compared to healthy controls, with a particular focus on distinguishing between the two clinical subtypes, postprandial distress syndrome (PDS) and epigastric pain syndrome (EPS). We hypothesize that specific miRNA signatures will differentiate FD patients with moderate to severe symptoms from controls, and that these profiles will further correlate with the severity of dyspeptic symptoms across PDS and EPS. Additionally, we seek to assess whether miRNA expression levels can stratify FD patients based on symptom severity, using the Functional Dyspepsia Symptom Diary - Total Symptom Score (FDSD-TSS) and functional dyspepsia symptom diary as a validated measure of symptom burden (12).

## 2 Materials and methods

This is a prospective observational case-control study of miRNA profiles from patients and controls that presented to our department between November 2023 and January 2025. The study was approved by the Ethics Committee of Iuliu Haţieganu University of Medicine and Pharmacy, Cluj-Napoca (Approval No. AVZ#243/27.09.2023).

### 2.1 Functional dyspepsia group

#### 2.1.1 Inclusion criteria

Eligible participants were men and women between 18 and 70 years of age who had a confirmed diagnosis of FD according to the Rome IV criteria. Each participant was required to have undergone an upper endoscopy within the past year with no significant findings that could explain their symptoms. Minor endoscopic findings like mild gastritis were permissible, provided that these did explain the patient’s dyspeptic symptoms. In cases where a patient had received H. pylori eradication therapy, they were only considered eligible once at least 6 months had passed since completing the eradication treatment. Active HP infection was excluded using rapid urease testing using AMA RUT EXPERT kit.

#### 2.1.2 Exclusion criteria

Patients were excluded if any medical or psychological condition might compromise safety or data integrity. This includes concurrent proton pump inhibitor (PPI) treatment, chronic opioid use, organic gastrointestinal diseases (such as inflammatory bowel disease or a history of gastrointestinal cancer), major systemic illnesses (like uncontrolled diabetes, thyroid disorders, or active malignancy unless in long-term remission), and prior surgeries that could alter gastrointestinal motility (excluding minor procedures like appendectomy). In addition, individuals with significant neuropsychiatric conditions or recent substance or alcohol abuse were not eligible, as were those whose symptoms predominantly suggested irritable bowel syndrome or reflux disease.

#### 2.1.3 Subject selection

Out of the 2234 gastroscopies performed in our department during the recruitment period, functional dyspepsia was diagnosed in 73 patients. Informed consent was obtained from 51 of these patients. However, 23 patients presented insufficient gastric juice volume, preventing them from participating in the study. Consequently, 28 patients were included in the final analysis.

### 2.2 Control group

The study enrolled 22 volunteer healthy men and women between 18 and 70 years of age, with no history of functional or organic gastrointestinal disorders. Gastroscopies were performed after informed consent was provided.

### 2.3 Gastric juice isolation

Gastroscopies were performed by 6 highly experienced gastroenterologists (T.S.B., S.P., A.C., C.S., F.R.). The gastroscope utilized in our department is the Olympus Evis Exera III 190. In all patients, a minimum of 10 milliliters of gastric juice was extracted in a sterile glass container using the aspiration system of the gastroscope, which was also sterilized beforehand.

Gastric juice samples were collected and immediately placed on ice, then aliquoted and stored at −80°C within an hour of collection. All samples remained at −80°C for a maximum of 6 months prior to RNA extraction to preserve miRNA integrity.

### 2.4 miRNA preparation

Gastric juice samples were centrifuged at × 2000 *g* for 30 min at 4°C. After centrifugation, the supernatant has been recovered and total RNA was extracted using the phenol-chloroform (TriReagent) method, according to the manufacturer’s protocol. The NanoDrop-1000 spectrophotometer (ThermoFischer Scientific, Waltham, MA, USA) was used to perform the RNA quantification and then the samples were diluted to a final concentration of 50 ng/μl for all the samples. For the selected miRNA(s), in the next step, TaqMan MicroRNA Transcription kit (ThermoFischer Scientific, Waltham, MA USA) and TaqMan microRNA assay (ThermoFischer Scientific, Waltham, MA USA) were used to reverse transcribed 50 ng of total RNA into cDNA according to the manufacturer’s protocol. miR-16 and U6 were used as housekeeping miRNAs. qRT-PCR was performed in duplicates for each sample in a 5 μL volume on a ViiA™ 7 System (Thermo Fischer Scientific, Waltham, MA, USA). ΔΔCT method was used to analyze the obtained CT values. To ensure analytical precision, all qRT-PCR reactions were run in technical duplicate for every sample. Intra-assay consistency was confirmed by the low variability between duplicate Ct values (typically with Ct standard deviations<0.5). We also assessed inter-assay reproducibility by re-running representative samples in separate qPCR runs. All batches were processed using the same lot of reagents and kits, with consistent RNA input amounts for each reaction. Furthermore, the qPCR machine was calibrated regularly, and all operators followed the same standard operating procedures.

### 2.5 Statistical analysis

Patient data were entered into a Microsoft Excel 2016 database. Statistical analyses were performed using R Commander version 4.5.0 (RCMDR) and Microsoft Excel (13). Categorical variables were summarized as absolute frequencies and percentages. Comparisons between groups for categorical data were conducted using the Chi-square test or Fisher’s exact test, depending on expected cell frequencies. Continuous variables were presented as means and standard deviations, respectively median and interquartile range for symptoms scores. Group differences for continuous variables were evaluated using either the Student’s *t*-test or the Mann–Whitney *U* test, depending on the distribution of the data. The association between miR levels and symptom categories was assessed using Kendall’s tau correlation coefficient. Normality of data distribution was tested using the Shapiro–Wilk test. A *p*-value < 0.05 was considered statistically significant.

## 3 Results

The clinical characteristics of the patents are detailed in Table 1. The studied population consisted of 28 patients with FD and 22 healthy controls. Of the 28 patients with FD, 14 were diagnosed with EPS, and 14 were suffering from PDS. When comparing the FD group (*n* = 28) with the HC group (*n* = 22), there were no statistically significant differences in age and sex. However, there was a statistically significant difference in weight (69.1 ± 13.0 vs. 80.6 ± 19.40) and height (165.8 ± 10.03 vs. 172.5 ± 14.09).

**TABLE 1:** Clinical characteristics of the study population.

Characteristics	FD (*n* = 28)	CONTROLS (*n* = 22)	*p*
Age (mean)	48.3 ± 15.08	48.5 ± 15.17	0.962
Age range	21–80	23–73	
Female (%)	18 (64.3%)	10 (45%)	0.183
Weight (kg, Mean + SD)	69.1 ± 13.03	80.6 ± 19.40	0.029*
Height (cm, Mean + SD, range)	165.8 ± 10.03	172.5 ± 14.09	0.02*
History of HP infection (*n*)	13		
History of infectious gastroenteritis (*n*)	7		
**FDSD item**			
Epigastric burning (median, IQR)	6 (4–8)		
Epigastric pain (median, IQR)	5 (5–7.25)		
Nausea (median, IQR)	3 (2–4)		
Bloating (Meadian, IQR)	0 (0–2)		
Stomach fulness (median, IQR)	1 (0–2)		
Early satiety (median, IQR)	1 (0–2.25)		
Burping/belching rating (median, IQR)	0 (0–1.25)		
Burping/belching bother (median, IQR)	0 (0–1.25)		
FDSD-TSS (median, IQR)	15 (12–17.5)		
Gastroscopy result (*n*)	28		
Normal gastroscopy (*n*)	17		
Mild gastritis (*n*)	9		

SD, standard deviation; IQR, interquartile range.

*p-value < 0.05.

### 3.1 Gastric juice miR levels in FD patients vs. healthy controls

**FIGURE 1:**
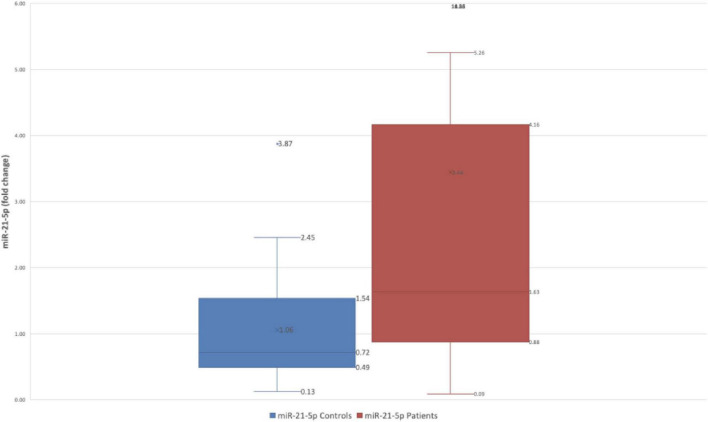
Difference in fold change between FD patients and controls for miR-21-5p.

Gastric juice levels of mir-21-5p differed significantly between FD cases and healthy controls (FD: 19.98 ± 91.56 fold-change vs. HC: 2.63 ± 3.30 fold-change *p* = 0.00774) Figure 1.

**FIGURE 2:**
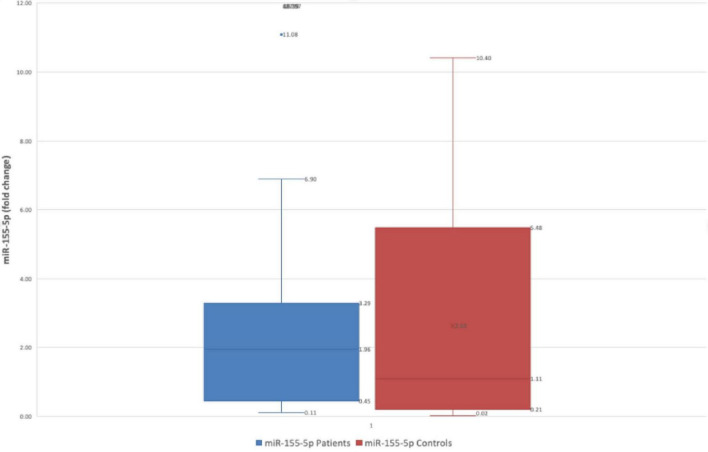
Difference in fold change between FD patients and controls for miR-155-5p.

Regarding miR-155-5p, a trend of decreased levels of gastric juice miR level was observed, with the average value in patients’ group was 2.63 ± 3.30 fold-change versus 19.98 ± 91.59 fold-change (*p* = 0.27- Mann Whitney test), but the result had no statistical significance Figure 2.

**FIGURE 3:**
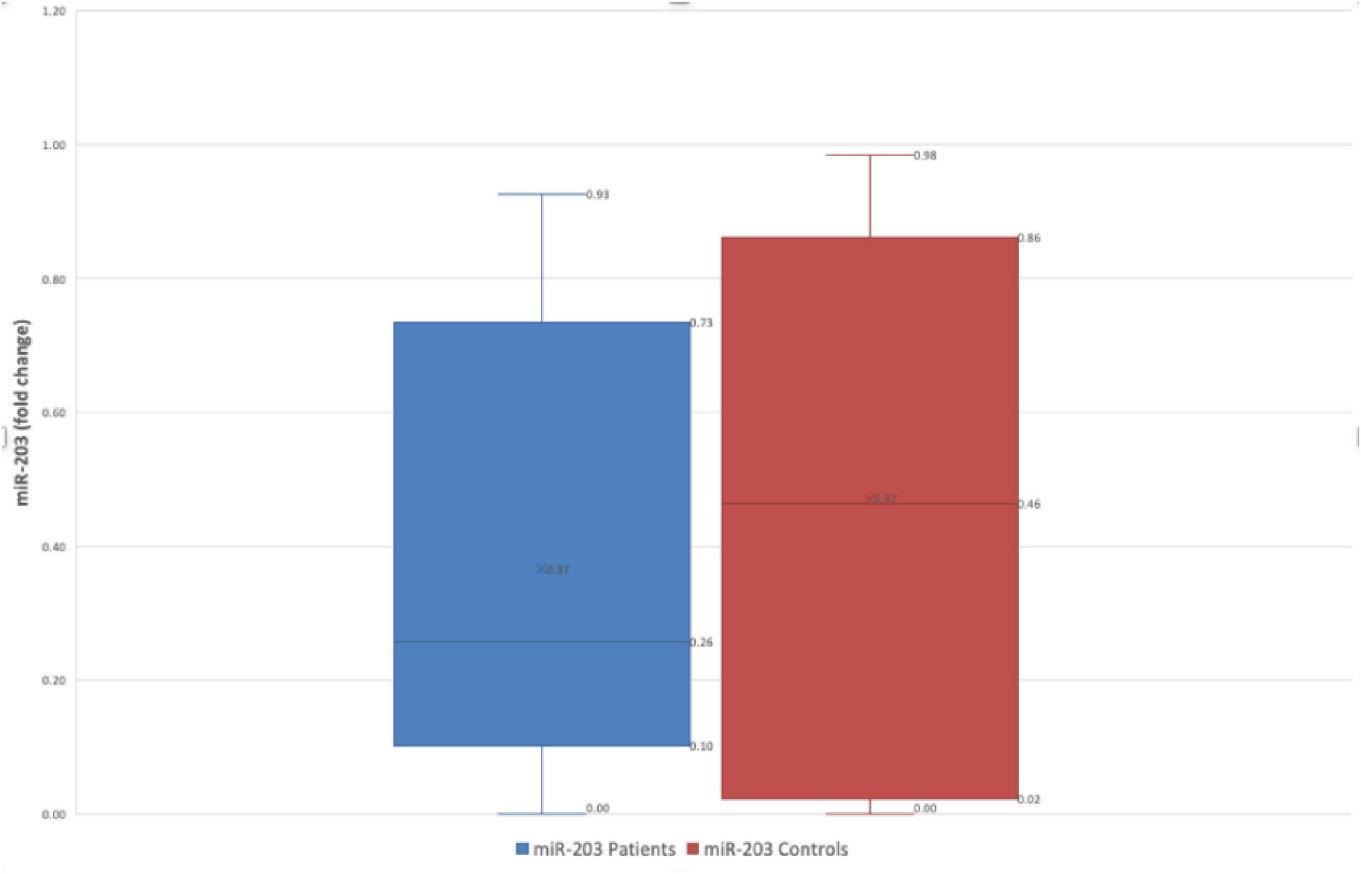
Difference in fold change between FD patients and controls for miR-203.

Conversely, miR-203 gastric juice level also showed a trend of decreased levels in FD patients vs. HC (FD: 0.36 ± 0.25 fold-change vs. HC: 0.47 ± 0.37 fold-change *p* = 0.272) Figure 3.

An outlier value was detected, yet the outcomes of the statistical analysis remained unchanged when removing this value.

We also attempted to analyze the expression of miR-933 and miR-708 in gastric juice, but ultimately the PCR amplification of these microRNAs was unsuccessful.

### 3.2 Comparing gastric juice miR levels in EPS vs. PDS patients

A total of 28 patients were included in the analysis, with 14 patients in the PDS group and 14 in the EPS group.

Expression of miR-155-5p was markedly higher in the PDS group (mean ± SD: 39.05 ± 128.99-fold-change; median: 3.24) than in the EPS group (0.92 ± 0.92-fold-change; median: 0.46). This difference was highly significant (*p* < 0.001).

Similarly, miR-21-5p levels were elevated in PDS patients (5.84 ± 5.19-fold-change; median: 3.86) relative to EPS patients (1.04 ± 0.84; median: 0.91), with *p* < 0.001.

In contrast, miR-203 expression was significantly lower in the PDS cohort (0.12 ± 0.13-fold-change; median: 0.11) compared with the EPS cohort (0.61 ± 0.27 fold-change; median: 0.73; *p* < 0.001). All between-group comparisons yielded *p*-values < 0.001, indicating robust differences in gastric juice miRNA profiles between PDS and EPS phenotypes.

### 3.3 Correlations between gastric juice miR levels and FDSD items

**FIGURE 4:**
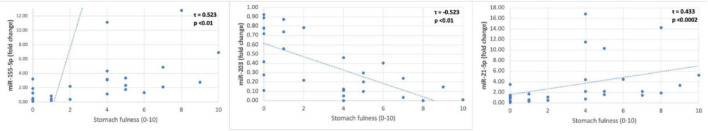
Gastric juice miR correlations with stomach fullness.

**FIGURE 5:**
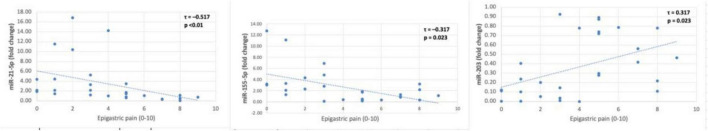
Gastric juice miR correlations with epigastric pain.

Significant associations were observed between gastric juice miR levels and the severity of dyspeptic symptoms (Kendall’s Tau B, *p* < 0.05). Notably, the direction of correlation differed between epigastric symptoms (pain and burning) and postprandial distress symptoms (fullness, early satiety, bloating) (Figures 4, 5).

Both epigastric burning and epigastric pain severities showed significant moderate inverse correlations with miR-155-5p and miR-21-5p, but positive correlations with miR-203. For example, epigastric burning intensity was strongly correlated with all three miRNAs: miR-155-5p (τ = −0.500, *p* < 0.001) and miR-21-5p (τ = −0.34, *p* = 0.014) were negatively correlated with burning severity, whereas miR-203 levels were positively correlated (τ = 0.500, *p* < 0.01). A similar pattern was observed for epigastric pain: higher pain scores corresponded to lower miR-155-5p (τ = −0.317, *p* = 0.023) and miR-21-5p (τ = –0.517, *p* < 0.01) levels, but higher miR-203 levels (τ = 0.317, *p* = 0.023).

In contrast to the epigastric symptoms, postprandial symptoms were positively correlated with miR-155-5p and miR-21-5p, and inversely with miR-203. For instance, stomach fullness severity was positively associated with miR-155-5p (τ = 0.523, *p* < 0.01) and miR-21-5p (τ = 0.433, *p* < 0.0002), while showing a significant negative correlation with miR-203 (τ = −0.523, *p* < 0.01). Early satiety demonstrated the same trend: miR-155-5p (τ = 0.426, *p* = 0.003) and miR-21-5p (τ = 0.398, *p* = 0.005) were directly correlated with satiety severity, whereas miR-203 was inversely correlated (τ = −0.426, *p* = 0.003). Bloating severity likewise had significant positive correlations with miR-155-5p (τ = 0.347, *p* = 0.015) and miR-21-5p (τ = 0.347, *p* = 0.015), and a negative correlation with miR-203 (τ = −0.347, *p* = 0.015).

Nausea did not show any significant correlation with the miRNA levels (all *p* > 0.05). Similarly, burping/belching frequency (self-reported rating) was not significantly associated with any miR. Finally, the composite FDSD-TSS was not significantly correlated with any individual miR level (*p* > 0.05 for miR-155-5p, miR-21-5p, and miR-203).

In subjects with a history of H. pylori infection, miR-203-fold change was significantly reduced compared to those without infection (Student’s *t* = −2.31, *p* = 0.029; confirmed by Mann–Whitney *U* = 53.0, *p* = 0.041), indicating a down-regulation of miR-203 in the HP-positive group.

**FIGURE 6:**
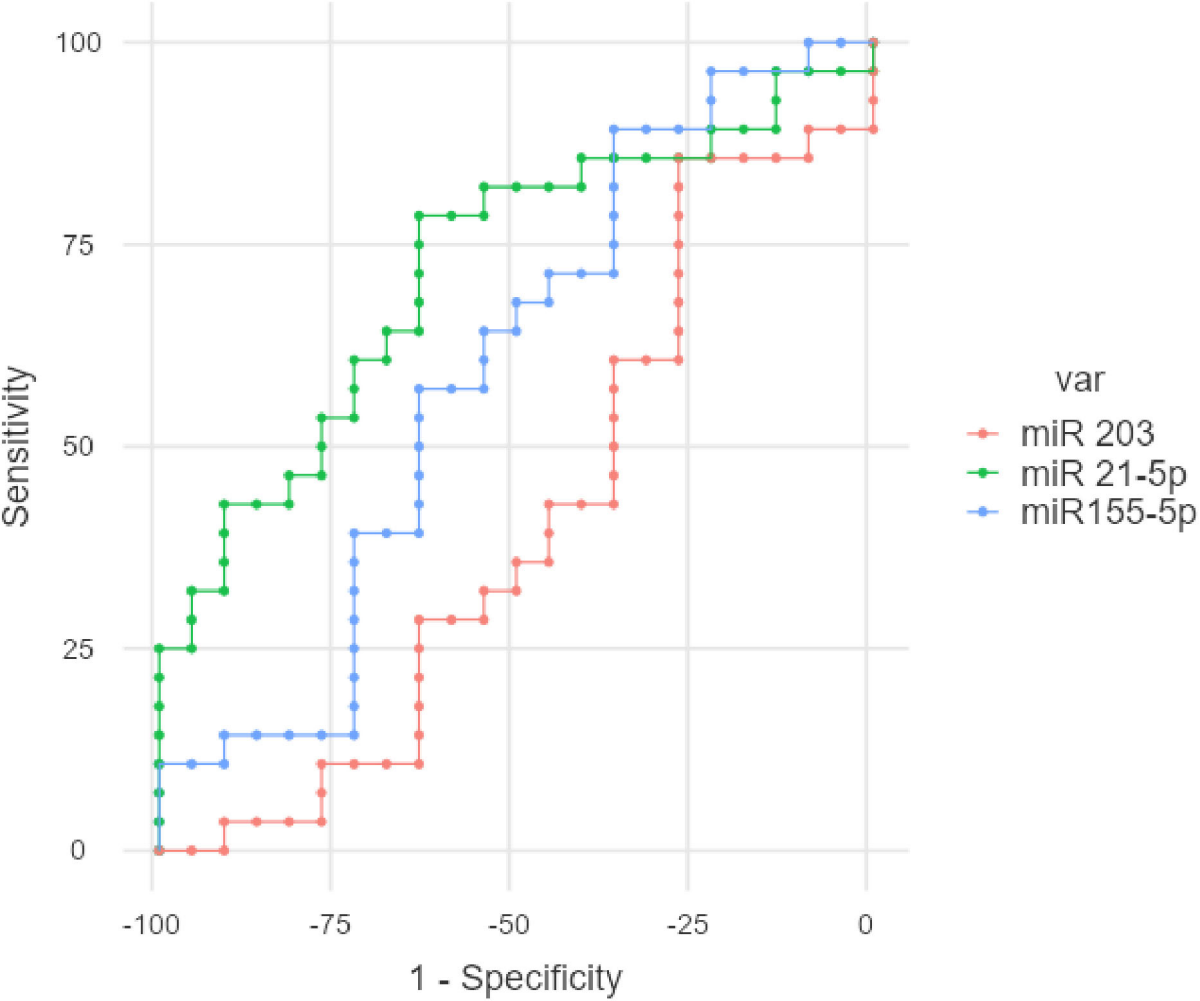
ROC analysis for the studied gastric juice miR in FD.

The ROC (Receiver Operating Characteristic) analysis was performed for miR-155-5p, miR-21-5p, and miR-203 to evaluate their individual diagnostic performance in differentiating FD patients from healthy controls. Among the three biomarkers, miR-21-5p showed the best diagnostic accuracy, with an AUC of 0.719, a sensitivity of 78.57%, and a specificity of up to 63.64% at the optimal cutpoint of 0.8435. In contrast, miR-155-5p demonstrated moderate performance (AUC: 0.593), with high sensitivity (89.29%) but low specificity (36.36%). miR-203 had the lowest discriminatory power (AUC: 0.407). The optimal cutpoints were determined using the Youden index (maximum sum of sensitivity and specificity). These results, which can be visualized in Figure 6.

## 4 Discussions

To our knowledge, this study has the largest cohort of FD patients that analysis gastric juice miR expression to this date. Our study demonstrates that patients with FD exhibit distinct gastric juice miRNA profiles compared to healthy controls, and even more pronounced differences between FD subtypes. FD patients had significantly elevated gastric miR-21-5p levels relative to controls (nearly eight-fold higher on average), whereas miR-155-5p and miR-203 levels showed nonsignificant downward trends in FD. Notably, the two FD clinical phenotypes, PDS and EPS, displayed inverse miRNA patterns. PDS patients had markedly higher gastric miR-21 and miR-155 levels but lower miR-203 than EPS patients (all *p* < 0.001). These miRNA differences were robust and mirrored symptom severity correlations: increased miR-21 and miR-155 associated with greater postprandial fullness and early satiety, whereas higher miR-203 correlated with worse epigastric pain and burning. This opposing correlation indicate that specific miRNAs may underlie the divergent pathophysiological mechanisms of PDS versus EPS.

Our findings both align with and diverge from previous research in gastric disease. miR-21, a well-known oncomiR, is overexpressed in gastric cancer tissue and blood, but intriguingly, prior work found that gastric juice levels of miR-21 are lower in gastric cancer patients compared to those with benign gastric conditions (14). miR-21 is also implicated in immune cell function, as it can drive macrophage polarization and cytokine release), miR-21 has been shown to affect intestinal barrier function: in an IBS model, inhibition of miR-21 caused increased intestinal epithelial permeability, indicating that miR-21 normally helps maintain barrier integrity (15). Our observation of elevated gastric juice miR-21 in FD, suggests that inflammatory or functional states can lead to higher luminal miR-21 release, whereas neoplastic conditions may sequester miR-21 in tissue or exosomes, reducing its gastric fluid presence.

In contrast, miR-155-5p did not differ significantly overall between FD and controls, but its high levels in PDS patients are noteworthy. miR-155 is a multifunctional miRNA with prominent roles in immune responses and carcinogenesis (16). miR-155 is induced by microbial stimuli and cytokines (e.g., in H. pylori gastritis) (17). Elevated miR-155-5p in FD gastric juice might thus reflect underlying mucosal immune activation. This microRNA can amplify Th1/Th17-type responses and promote pro-inflammatory cytokine production, potentially leading to subtle inflammation in the gastroduodenal mucosa (18). Such inflammation is known to sensitize afferent nerves and could underlie the visceral hypersensitivity observed in FD. The fact that only the PDS subgroup (and not EPS) showed elevated miR-155 suggests a subset of FD may involve an inflammatory component or post-infectious change that activates this pathway. Meanwhile, EPS patients, whose symptoms are more pain-centered, had uniformly low miR-155, aligning with a mechanism less driven by active inflammation and more by neural sensitivity. To our knowledge, this is the first report of altered miR-155 in FD subtypes. It extends prior work by Tanaka et al., who profiled exosomal miRNAs in FD and identified a different miRNA (exosomal miR-933) as significantly downregulated in FD vs. controls (11). Interestingly, Tanaka’s study also found that lower exosomal miR-933 levels correlated with higher dyspepsia symptom scores and more frequent epigastric pain (11). Our results similarly demonstrate symptom-linked miRNA changes, reinforcing the concept that miRNA expression is functionally relevant in FD. Unlike Tanaka’s focus on downregulated miRNAs, our data reveal upregulation of certain miRNAs (miR-21, miR-155) in FD, possibly due to measuring total gastric juice miRNAs rather than only exosomal fractions.

miR-203 emerged in our study as an inverse marker to miR-21/miR-155. miR-203a has been implicated in gastric mucosal biology; for example, *H. pylori* infection significantly down-regulates miR-203 in gastric tissues, which can lead to increased expression of pro-inflammatory or proliferative target genes. In FD, changes in miR-203a could reflect mucosal stress responses or an attempt to maintain epithelial integrity. If miR-203a is dysregulated, it might disturb the balance of epithelial renewal and immune tolerance in the stomach, subtly contributing to symptom generation (19). EPS patients, many of whom had no H. pylori history, maintained higher miR-203, which in our data correlated with greater pain intensity.

We normalized qPCR data to miR-16 and U6, which were selected based on prior reports of stable expression in gastric specimens (20). The use of two reference genes was intended to improve accuracy, but an ideal strategy would be to validate reference genes in our gastric juice samples or use multiple references (21). Future studies might employ alternate normalizers or global normalization to confirm our findings.

In our study, FD patients had lower weight and height than controls. This raises the possibility of confounding, larger individuals, controls, might have different gastric juice characteristics than smaller individuals. We did not control for this, which is a limitation. We recommend future research match participants by BMI or adjust for it, to ensure that observed miRNA differences are truly due to FD status and not body size.

In this pilot study, we performed primarily univariate analyses to compare miRNA levels between groups. We recognize that multivariate models (e.g., combining all three miRNAs in a logistic regression or adjusting for covariates) could further strengthen the analysis. We did not include such models in the current manuscript due to the limited sample size (which could lead to overfitting) (22).

This was a single-center study with a small sample size, which may limit generalizability. The smaller sample size indicates that the present study cannot draw definitive conclusions. Future studies with greater sample sizes are required to reliably establish these miRNAs as biomarkers. Another limitation of the present study was the lack of an internal validation cohort. The present results need to be further validated in subsequent studies for validation and generalizability.

We did not control for or document potentially influential pre-analytical variables such as recent dietary intake, medication use (except for PPIs) or lifestyle factors (e.g., smoking, alcohol use, stress). These unmeasured variables may alter gastric juice composition and could impact extracellular miRNA expression levels. Future studies should include standardized dietary or medication washout protocols and gather comprehensive lifestyle histories to evaluate and control for these potential confounders. Furthermore, for gastric juice miRNAs to be translated into clinical biomarkers, protocols must be developed to ensure reproducibility under real-world clinical conditions. Larger studies are needed to confirm the stability of these biomarkers. The absence of tissue biopsy and correlation with miR values is another possible limitation and further studies could validate a possible correlation between mucosal histopathology and related biomarkers.

These results highlight the potential of gastric juice miRNAs as non-invasive biomarkers for FD. The ability to differentiate PDS from EPS using molecular signatures is especially valuable. Currently, FD subtyping relies purely on symptom questionnaires, which can be subjective and overlapping. In our study, gastric juice miR-21, miR-155, and miR-203 distinguished PDS vs. EPS with high statistical significance. Recent advancements in miR detection have positioned them as promising candidates for use as biomarkers in a range of diseases (23). If validated in larger cohorts, a panel of these miRNAs could aid clinicians in objectively subclassifying FD patients. It is also noteworthy that gastric juice collection is relatively simple during routine endoscopy. This makes serial sampling feasible. In the future, miRNA profiles might be tracked over time or after interventions.

## 5 Conclusion

In summary, this study provides new evidence that gastric juice miRNAs, specifically miR-21, miR-155, and miR-203, might be dysregulated in FD and differ strikingly between PDS and EPS subtypes, and might be correlating with dyspepsia symptom severity. Gjuice miRNAs hold promise as biomarkers for improving FD diagnosis and subtype stratification. They also open new avenues to understand FD’s pathophysiology, potentially linking immune, microbial, and neuromuscular factors through gene regulatory networks. Further research should build on these findings to validate diagnostic performance and to reveal the mechanistic roles of these miRNAs in the gut–brain axis of functional dyspepsia.

## Data Availability

The authors acknowledge that the data presented in this study must be deposited and made publicly available in an acceptable repository, prior to publication. Frontiers cannot accept a manuscript that does not adhere to our open data policies.
